# Hypoglycemia and Long-Term Cardiovascular Outcomes in Diabetes Mellitus: A Systematic Review of Prognostic Evidence

**DOI:** 10.7759/cureus.109434

**Published:** 2026-05-22

**Authors:** Fawad Haroon, Nabila N Anika, Joshua James, Musa Khan, Bhavna Singla, Shivam Singla, Sunita Kumawat, Asad Talha

**Affiliations:** 1 Internal Medicine, TidalHealth Peninsula Regional, Salisbury, USA; 2 Internal Medicine, Holy Family Red Crescent Medical College Hospital, Dhaka, BGD; 3 Internal Medicine, Rehman Medical College, Peshawar, PAK; 4 Internal Medicine, Belarusian State Medical University, Minsk, BLR; 5 Internal Medicine, Erie County Medical Center Health Campus, Buffalo, USA; 6 Internal Medicine, St. Francis Medical Center, Lynwood, USA; 7 Internal Medicine, Jinnah Medical and Dental College, Karachi, PAK

**Keywords:** all-cause mortality, cardiovascular mortality, cardiovascular outcomes, diabetes mellitus, glycemic control, hypoglycemia, major adverse cardiovascular events, severe hypoglycemia

## Abstract

Hypoglycemia is a frequent complication of glucose-lowering therapy in diabetes mellitus and has been increasingly linked to adverse cardiovascular outcomes. This systematic review aimed to evaluate the association between hypoglycemic episodes and long-term cardiovascular outcomes, including cardiovascular mortality, major adverse cardiovascular events (MACE), and all-cause mortality. A comprehensive search of PubMed/Medical Literature Analysis and Retrieval System Online (MEDLINE), Scopus, and Web of Science was conducted for studies published between January 2000 and December 2024. Randomized trial-derived analyses and observational cohort studies assessing hypoglycemia as an exposure and reporting cardiovascular or mortality outcomes in adults with diabetes were included. Seven studies met the inclusion criteria. Across the included studies, severe hypoglycemia was consistently associated with an increased risk of cardiovascular events and mortality. In contrast, non-severe hypoglycemia showed less consistent associations and appeared to confer risk primarily at higher frequencies. Temporal analyses suggested an elevated risk of adverse outcomes shortly after hypoglycemic episodes, while some studies demonstrated dose-response relationships. However, interpretation remains complex, as hypoglycemia often occurs in patients with higher baseline risk, raising the possibility that it may act as both a contributing factor and a marker of vulnerability. In conclusion, severe hypoglycemia is associated with increased cardiovascular risk and mortality in patients with diabetes. These findings support the importance of individualized glycemic management strategies that minimize hypoglycemia while maintaining effective metabolic control.

## Introduction and background

Diabetes mellitus is a major global health burden and a leading contributor to cardiovascular morbidity and mortality. Despite advances in glucose-lowering therapies and risk factor modification, individuals with diabetes continue to experience a substantially elevated risk of major adverse cardiovascular events (MACE) and premature death [1,2]. Glycemic control remains a central component of diabetes management, with substantial evidence supporting the reduction of microvascular complications. However, the relationship between glycemic targets, treatment intensity, and long-term cardiovascular outcomes is complex, particularly in the context of treatment-related adverse effects such as hypoglycemia [3].

Hypoglycemia, especially when severe, represents one of the most clinically significant and potentially life-threatening complications of glucose-lowering therapy. It has been increasingly recognized not only as an acute metabolic disturbance but also as a potential contributor to adverse cardiovascular outcomes [4]. Mechanistically, hypoglycemia may induce a cascade of physiological responses, including sympathoadrenal activation, pro-arrhythmic effects, endothelial dysfunction, and pro-inflammatory and pro-thrombotic states. These pathophysiological changes provide a plausible biological basis for the observed associations between hypoglycemic episodes and cardiovascular events [5,6]. At the same time, hypoglycemia frequently occurs in individuals with advanced disease, comorbid conditions, and higher baseline cardiovascular risk, raising important questions regarding whether it serves as a causal factor or a marker of underlying vulnerability [7].

Large randomized controlled trials and observational cohort studies have explored the relationship between hypoglycemia and cardiovascular outcomes, yielding important but sometimes heterogeneous findings. Several studies have demonstrated significant associations between severe hypoglycemia and increased risks of cardiovascular events, cardiovascular mortality, and all-cause mortality. In contrast, the impact of non-severe hypoglycemia appears less consistent and may depend on the frequency and clinical context of episodes. Furthermore, temporal analyses suggest that the risk of adverse outcomes may be heightened in the period immediately following hypoglycemic events, while dose-response relationships observed in some cohorts further complicate causal interpretation. These variations underscore the need for a careful synthesis of the available evidence, with attention to study design, patient populations, and definitions of hypoglycemia [8].

The objective of this systematic review is to comprehensively evaluate the association between hypoglycemic episodes and long-term cardiovascular outcomes, including cardiovascular mortality, in individuals with diabetes mellitus. By synthesizing evidence from randomized controlled trials and observational studies, this review aims to clarify the strength, consistency, and clinical relevance of this relationship, while critically appraising whether hypoglycemia functions as an independent risk factor or a marker of heightened cardiovascular vulnerability.

## Review

Materials and methods

Study Design and Reporting Framework

This systematic review was conducted in accordance with the Preferred Reporting Items for Systematic Reviews and Meta-Analyses (PRISMA) 2020 guidelines [9]. The methodological approach was structured to ensure transparency, reproducibility, and comprehensive synthesis of the available evidence. The review was designed to evaluate hypoglycemia as a prognostic exposure and its association with subsequent cardiovascular outcomes and mortality in individuals with diabetes mellitus. A formal protocol for this review was not prospectively registered in the International Prospective Register of Systematic Reviews (PROSPERO) or any other public registry, which should be considered a methodological limitation.

Research Question and the Population, Intervention/Exposure, Comparator, and Outcome (PICO) Framework

The research question was formulated using the PICO framework [10]. The population included adult patients with type 1 or type 2 diabetes mellitus. The exposure of interest was hypoglycemia, with particular emphasis on severe hypoglycemic episodes, although studies evaluating non-severe hypoglycemia were also considered when relevant. The comparator consisted of patients without hypoglycemic episodes or with a lower frequency of such events. The primary outcomes included cardiovascular mortality, MACE, and all-cause mortality. This framework guided the development of the search strategy, eligibility criteria, and data extraction process.

Literature Search Strategy

A comprehensive literature search was conducted across multiple electronic databases, including PubMed/Medical Literature Analysis and Retrieval System Online (MEDLINE), Scopus, and Web of Science. The search covered studies published from January 2000 to December 2024, reflecting the time period in which major cardiovascular outcome trials and large cohort studies on hypoglycemia have been reported. The search strategy combined Medical Subject Headings (MeSH) terms and free-text keywords related to hypoglycemia and cardiovascular outcomes. Key search terms included “hypoglycemia,” “severe hypoglycemia,” “cardiovascular mortality,” “cardiovascular events,” “major adverse cardiovascular events,” and “diabetes mellitus.” Boolean operators were applied to refine the search, using combinations such as “hypoglycemia AND cardiovascular mortality,” “severe hypoglycemia AND cardiovascular events,” and “diabetes AND hypoglycemia AND mortality.” Reference lists of included studies were also screened to identify additional relevant articles.

Eligibility Criteria

Studies were selected based on predefined inclusion and exclusion criteria aligned with the objectives of the review. Eligible studies included randomized controlled trials, post hoc analyses of cardiovascular outcome trials, and observational cohort studies that evaluated the association between hypoglycemia and cardiovascular outcomes or mortality in adult patients with diabetes. Studies were required to report clinically relevant outcomes, including cardiovascular mortality, MACE, or all-cause mortality, and to provide effect estimates such as hazard ratios or relative risks. Both severe and non-severe hypoglycemia were considered, provided that definitions were clearly described.

Importantly, studies were included if hypoglycemia was analyzed as an independent prognostic exposure, either as a predefined exposure variable (as in observational cohort studies) or as a time-dependent or treatment-emergent variable in post hoc analyses of randomized controlled trials, provided that appropriate statistical modeling was used to evaluate its association with outcomes.

Exclusion criteria included studies involving pediatric populations, case reports, editorials, narrative reviews, and studies lacking clear outcome measures or insufficient statistical analysis. Studies that did not analyze hypoglycemia as an independent prognostic variable or failed to report cardiovascular or mortality outcomes were also excluded. To ensure analytical consistency and relevance, studies focusing solely on short-term biochemical outcomes without clinically relevant endpoints were not considered.

Study Selection Process

All identified records were screened initially by title and abstract to assess relevance. Full-text articles were then reviewed for eligibility based on the predefined criteria. Studies that met the inclusion criteria were selected for qualitative synthesis. Preference was given to large-scale randomized trials, post hoc analyses of cardiovascular outcome trials, and high-quality cohort studies to ensure that the included evidence was both clinically meaningful and methodologically robust.

Data Extraction and Synthesis

Data were extracted using a standardized approach, focusing on key study characteristics including study design, population size, patient characteristics, definitions of hypoglycemia, duration of follow-up, and reported cardiovascular and mortality outcomes. Particular attention was given to effect estimates, temporal relationships, and dose-response patterns where available. Given the heterogeneity in study design, definitions, and outcome measures, a quantitative meta-analysis was not performed. Instead, a structured narrative synthesis was conducted to integrate findings across studies and to identify consistent patterns and clinically relevant insights.

Risk of Bias Assessment

The methodological quality of the included studies was assessed using the Quality In Prognosis Studies (QUIPS) tool [11], which is appropriate for evaluating studies where the exposure of interest functions as a prognostic factor. Domains assessed included study participation, attrition, prognostic factor measurement, outcome measurement, confounding, and statistical analysis. Overall, most studies were judged to have a low to moderate risk of bias, with residual confounding identified as the most consistent limitation across both randomized trial-derived analyses and observational cohorts.

Results

Study Selection Process

**Figure 1:**
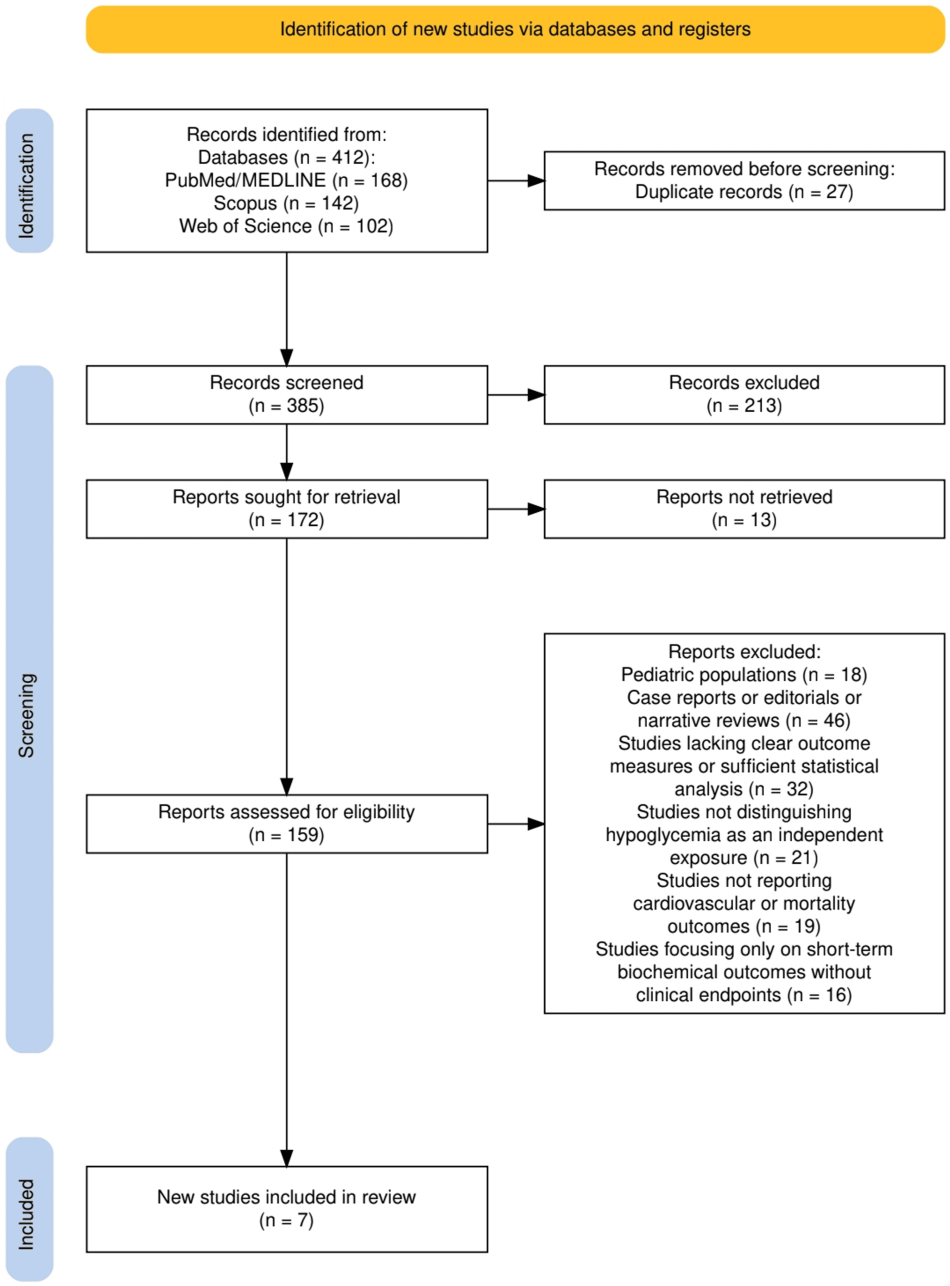
PRISMA flow diagram illustrating the study selection process from identification to inclusion. PRISMA: Preferred Reporting Items for Systematic Reviews and Meta-Analyses; MEDLINE: Medical Literature Analysis and Retrieval System Online

A total of 412 records were initially identified through database searching, including PubMed/MEDLINE, Scopus, and Web of Science. After removal of 27 duplicate records, 385 studies underwent title and abstract screening, of which 213 were excluded based on lack of relevance. A total of 172 reports were sought for retrieval, with 13 reports not retrieved, leaving 159 studies for full-text assessment. Of these, 152 studies were excluded for predefined reasons, including pediatric populations, non-original study designs such as case reports and narrative reviews, lack of clear outcome measures or adequate statistical analysis, failure to define hypoglycemia as an independent exposure, absence of cardiovascular or mortality outcomes, and focus on short-term biochemical endpoints without clinical relevance. Ultimately, seven studies met the inclusion criteria and were included in the qualitative synthesis. The detailed study selection process is illustrated in Figure 1.

Characteristics of the Selected Studies

The included studies comprised a combination of large randomized controlled trial-derived analyses and observational cohort studies, providing a comprehensive and complementary evidence base for evaluating the relationship between hypoglycemia and cardiovascular outcomes [8, 12-17]. Several studies were based on established cardiovascular outcome trials involving patients with type 2 diabetes at high cardiovascular risk, with follow-up durations ranging from approximately 3.5 to 10.4 years and the use of adjudicated clinical endpoints. Notably, the studies by Zinman et al. and Heller et al. were separate post hoc analyses derived from the same LEADER (Liraglutide Effect and Action in Diabetes: Evaluation of Cardiovascular Outcome Results) trial population [12, 15], examining different hypoglycemia-related exposures and outcome associations. In parallel, large-scale observational cohorts, including nationwide and registry-based studies, contributed real-world data with longer follow-up periods and the ability to assess temporal and dose-response relationships. Across studies, hypoglycemia was primarily defined as severe episodes requiring external assistance, although some studies also incorporated non-severe events based on biochemical thresholds or self-monitoring. The primary outcomes consistently included MACE, cardiovascular mortality, and all-cause mortality. Hypoglycemia was evaluated as a prognostic exposure across both randomized trial-derived analyses and observational cohorts. Variability in patient populations, baseline risk profiles, and definitions of hypoglycemia introduced a degree of heterogeneity, but overall, the included studies provided a robust and clinically relevant dataset. The detailed characteristics of the selected studies are presented in Table 1.

**Table 1:** Summary of study designs, populations, hypoglycemia definitions, and cardiovascular outcomes in the included studies. ADVANCE: Action in Diabetes and Vascular Disease: Preterax and Diamicron MR Controlled Evaluation; CV: cardiovascular; HR: hazard ratio; ICD-10: International Classification of Diseases, Tenth Revision; LEADER: Liraglutide Effect and Action in Diabetes: Evaluation of Cardiovascular Outcome Results; MACE: major adverse cardiovascular events; MI: myocardial infarction; NSHE: non-severe hypoglycemic events; ORIGIN: Outcome Reduction with Initial Glargine Intervention; RCT: randomized controlled trial; T2DM: type 2 diabetes mellitus; VADT: Veterans Affairs Diabetes Trial

Study (year)	Design	Population (n)	Hypoglycemia definition	Outcome(s)	Follow-up	Key findings
Zinman et al. (2018): LEADER [12]	RCT (post hoc analysis)	9,340 patients with T2DM (high CV risk)	Severe hypoglycemia (requiring assistance; modeled as a time-dependent covariate)	MACE, CV death, all-cause mortality	3.5–5 years	Severe hypoglycemia was associated with increased risk of MACE, CV death, and all-cause mortality, with the highest risk observed shortly after hypoglycemic episodes
Yun et al. (2019): Nationwide cohort [8]	Population-based retrospective cohort	1,568,097 patients with T2DM	Severe hypoglycemia (ICD-10 codes; frequency-based classification)	MI, stroke, heart failure, all-cause mortality	Mean follow-up: 5.7 years	Severe hypoglycemia was associated with increased risk of CV events and all-cause mortality, demonstrating a dose–response relationship and the highest risk within the first year following episodes
Davis et al. (2018): VADT [13]	RCT (post hoc analysis)	1,791 patients with T2DM (high CV risk veterans)	Severe hypoglycemia (recent episodes within three months)	CV events, CV mortality, all-cause mortality	Mean follow-up: 5.6 years	Recent severe hypoglycemia was associated with increased risk of CV events, cardiovascular mortality, and all-cause mortality, with a higher risk observed in patients with greater baseline CV risk
Cha et al. (2016) [14]	Prospective cohort	1,260 patients with T2DM	Severe hypoglycemia (≥1 episode requiring assistance)	CV death, all-cause mortality	Mean follow-up: 10.4 years	Severe hypoglycemia was strongly associated with increased CV mortality (HR ~6.34) and all-cause mortality
Heller et al. (2022): LEADER (NSHE analysis) [15]	RCT (post hoc analysis)	LEADER cohort (patients with T2DM and high CV risk)	Non-severe hypoglycemia (glucose <3.1 mmol/L) and severe hypoglycemia (requiring assistance)	MACE, CV death, all-cause mortality	3.5–5 years	High frequency of non-severe hypoglycemia (≥12/year) was associated with increased risk of MACE, CV death, and all-cause mortality, and predicted future severe hypoglycemia
Mellbin et al. (2013): ORIGIN [16]	RCT (post hoc analysis)	12,537 patients with dysglycemia and high CV risk	Severe hypoglycemia (requiring assistance or glucose ≤36 mg/dL) and non-severe hypoglycemia (≤54 mg/dL)	MACE, CV death, all-cause mortality	Mean follow-up: 6.2 years	Severe hypoglycemia was associated with increased CV death (HR ~1.71), mortality, and CV events, whereas non-severe hypoglycemia was not associated after adjustment
Zoungas et al. (2010): ADVANCE [17]	RCT (post hoc analysis)	11,140 patients with T2DM	Severe hypoglycemia (requiring assistance)	Major macrovascular events, CV death, all-cause mortality	5 years	Severe hypoglycemia was associated with increased risk of macrovascular events (HR ~2.88), CV death (HR ~2.68), and all-cause mortality (HR ~2.69), although the association may reflect underlying vulnerability rather than direct causality

Quality Assessment

The methodological quality of the included studies was assessed using the QUIPS tool, which is appropriate for evaluating hypoglycemia as a prognostic exposure. Overall, the studies demonstrated low to moderate risk of bias across most domains, reflecting generally robust study designs and outcome ascertainment. Randomized trial-derived analyses showed strengths in participant selection, follow-up, and adjudicated cardiovascular outcomes, while observational cohort studies contributed valuable real-world evidence with large sample sizes and extended follow-up durations. The most consistent limitation across studies was the potential for residual confounding, as hypoglycemia is more likely to occur in patients with advanced disease, greater comorbidity burden, and higher baseline cardiovascular risk. Variability in the definition and measurement of hypoglycemia, particularly for non-severe events, also introduced some degree of measurement bias. Despite these limitations, the overall quality of evidence was considered acceptable for qualitative synthesis, with findings demonstrating reasonable internal consistency across studies. A detailed summary of the risk of bias assessment is presented in Table 2.

**Table 2:** Risk of bias assessment of included studies using the QUIPS tool across key methodological domains. ADVANCE: Action in Diabetes and Vascular Disease: Preterax and Diamicron MR Controlled Evaluation; LEADER: Liraglutide Effect and Action in Diabetes: Evaluation of Cardiovascular Outcome Results; NSHE: non-severe hypoglycemic events; ORIGIN: Outcome Reduction with Initial Glargine Intervention; QUIPS: Quality In Prognosis Studies; RCT: randomized controlled trial; RoB: Risk of Bias; VADT: Veterans Affairs Diabetes Trial

Study (year)	Study type in this review	Suitable RoB tool	Study participation	Study attrition	Prognostic factor measurement	Outcome measurement	Study confounding	Statistical analysis and reporting	Overall risk of bias
Zinman et al. (2018): LEADER [12]	Post hoc prognostic analysis of RCT	QUIPS	Low	Low	Low	Low	Moderate	Low	Moderate
Yun et al. (2019): Nationwide cohort [8]	Population-based retrospective cohort	QUIPS	Low	Low	Low	Low	Moderate	Low	Moderate
Davis et al. (2018): VADT [13]	Post hoc prognostic analysis of RCT	QUIPS	Moderate	Moderate	Low	Low	Moderate	Low	Moderate
Cha et al. (2016): Vincent registry [14]	Prospective cohort	QUIPS	Low	Low	Low	Low	Moderate	Moderate	Moderate
Heller et al. (2022): LEADER (NSHE analysis) [15]	Post hoc prognostic analysis of RCT	QUIPS	Low	Low	Low	Low	Moderate	Low	Moderate
Mellbin et al. (2013): ORIGIN [16]	Post hoc prognostic analysis of RCT	QUIPS	Low	Low	Moderate	Moderate	Moderate	Low	Moderate
Zoungas et al. (2010): ADVANCE [17]	Post hoc prognostic analysis of RCT	QUIPS	Low	Low	Moderate	Low	Moderate	Low	Moderate

Discussion

What Does the Evidence Actually Show?

Across the included studies, a consistent association emerges between severe hypoglycemia and adverse cardiovascular outcomes, including cardiovascular mortality, although the magnitude and context of this relationship vary. Large randomized trial-derived analyses, including those by Zoungas et al. (ADVANCE (Action in Diabetes and Vascular Disease: Preterax and Diamicron MR Controlled Evaluation)) [17], Mellbin et al. (ORIGIN (Outcome Reduction with Initial Glargine Intervention)) [16], Zinman et al. (LEADER) [12], and Davis et al. (VADT (Veterans Affairs Diabetes Trial)) [13], collectively demonstrate that severe hypoglycemia is associated with increased risks of MACE and death. This signal is further reinforced by observational data, such as the nationwide cohort reported by Yun et al. [8], which highlights dose-response relationships and temporal patterns. In contrast, the impact of non-severe hypoglycemia appears less consistent, with some analyses, including those by Mellbin et al. [16], showing no significant association after adjustment, while others, such as Heller et al. [15], suggest increased risk only at higher episode frequencies. These findings indicate convergence across diverse study designs, yet also underscore heterogeneity in exposure definitions, patient populations, and effect sizes. An additional consideration is glucose variability, which may both predispose patients to hypoglycemic episodes and independently contribute to cardiovascular risk through oxidative stress, endothelial dysfunction, autonomic instability, and inflammatory pathways. In this context, hypoglycemia may represent one component of a broader dysglycemic profile, supporting the importance of individualized glycemic strategies that address not only mean glucose control but also excessive glycemic fluctuations. Taken together, hypoglycemia emerges as a reproducible clinical signal linked to cardiovascular risk. Within this context, hypoglycemia should be interpreted as a prognostic exposure rather than a primary etiologic factor, and the available evidence does not establish a definitive causal relationship, with residual confounding inherent to prognostic analyses.

Mechanistic Integration

Several biological mechanisms provide a plausible framework linking hypoglycemia to adverse cardiovascular outcomes. Acute hypoglycemia triggers sympathoadrenal activation, resulting in catecholamine release that can induce tachycardia, vasoconstriction, and increased myocardial oxygen demand. This state may contribute to ischemia in individuals with underlying cardiovascular disease [6]. In addition, hypoglycemia has been associated with electrophysiological changes, including QT interval prolongation, which may predispose to malignant arrhythmias and sudden cardiac death, a finding that aligns with the increased risk of arrhythmic death observed in Mellbin et al. (ORIGIN) [16]. Beyond these acute effects, hypoglycemia may promote a prothrombotic and inflammatory milieu, characterized by platelet activation, endothelial dysfunction, and increased circulating inflammatory markers. These processes can further destabilize atherosclerotic plaques and impair vascular integrity. While these mechanisms collectively support the biological plausibility of a link between hypoglycemia and cardiovascular events, they remain largely inferential in clinical populations and do not, in isolation, establish a direct causal pathway [18].

Temporal and Dose-Response Insights

One of the most informative patterns across the included literature is the combined presence of temporal and dose-response signals, which adds interpretive depth beyond a simple statement of association. In Zinman et al. (LEADER) [12] and Davis et al. (VADT) [13], the risk of adverse cardiovascular outcomes and death was greatest in the period shortly after severe hypoglycemia, suggesting that such episodes may act as potential proximate stressors in patients with underlying cardiovascular susceptibility. In contrast, the nationwide cohort reported by Yun et al. [8] demonstrated a graded increase in cardiovascular outcomes and mortality with rising frequency of severe hypoglycemic episodes, which supports a dose-response relationship and strengthens the argument that recurrent hypoglycemia identifies a subgroup at progressively higher risk. At the same time, Zoungas et al. (ADVANCE) [17] did not find a clear cumulative relationship between repeated episodes and vascular outcomes or death, indicating that frequency alone may not fully account for subsequent risk across all settings. Taken together, these findings support a more nuanced interpretation in which hypoglycemia may function as a short-term trigger of adverse events in vulnerable patients, while also serving as a longer-term marker of underlying fragility, treatment complexity, and cardiovascular susceptibility.

Causality Versus Marker of Vulnerability

A central interpretive challenge in this literature is whether hypoglycemia should be understood primarily as a direct contributor to cardiovascular harm or as a clinical marker of patients who are already at elevated risk. The direct causal hypothesis is supported by several observations, including the temporal proximity between severe hypoglycemia and cardiovascular events in Zinman et al. (LEADER) [12] and Davis et al. (VADT) [13], the biological plausibility of autonomic, arrhythmic, inflammatory, and hemodynamic mechanisms, and the dose-response pattern observed by Yun et al. [8]. However, the marker hypothesis remains equally compelling. In Zoungas et al. (ADVANCE) [17], severe hypoglycemia was associated not only with vascular outcomes but also with a broader range of nonvascular adverse events, suggesting that it may reflect a state of generalized vulnerability rather than a pathway specific to cardiovascular injury. Across studies, hypoglycemia was more common in individuals with longer diabetes duration, insulin use, chronic kidney disease, heart failure, and other markers of advanced disease, all of which independently increase cardiovascular risk. In addition, the predominance of post hoc and observational analyses introduces the possibility of residual confounding and reverse causation. A balanced interpretation is therefore that the relationship is likely bidirectional. Hypoglycemia may both reflect pre-existing frailty and disease burden and, in susceptible individuals, contribute to the precipitation of subsequent cardiovascular events and death [19].

Clinical Implications

From a clinical perspective, the available evidence suggests that hypoglycemia should be regarded not merely as an undesirable adverse effect of treatment, but also as a meaningful prognostic signal that may identify patients at heightened cardiovascular risk. This is particularly relevant in older adults and in individuals with chronic kidney disease, established cardiovascular disease, long-standing diabetes, or insulin-treated disease, all of whom appear more vulnerable to both hypoglycemia and adverse cardiovascular outcomes. Importantly, these findings do not support abandoning glycemic control as a therapeutic goal. Rather, they reinforce the importance of individualized glycemic targets, careful treatment selection, and avoidance of overtreatment in patients with high competing risks. Consistent with contemporary American Diabetes Association (ADA) and the European Association for the Study of Diabetes (EASD) consensus recommendations, treatment de-intensification or simplification should be considered in selected high-risk patients when the risks of hypoglycemia or treatment burden outweigh the expected benefits of intensive glycemic control. The practical implication is not therapeutic nihilism, but a more balanced and patient-centered approach in which efforts to improve glycemic control are pursued alongside strategies to minimize severe and recurrent hypoglycemia [20].

Strengths

This review has several strengths that enhance its interpretive value. First, it synthesizes evidence from both large randomized trial-derived analyses and large observational cohorts, allowing assessment of consistency across study designs and clinical settings. Studies such as Zoungas et al. (ADVANCE) [17], Mellbin et al. (ORIGIN) [16], Zinman et al. (LEADER) [12], and Davis et al. (VADT) [13] provide high-quality adjudicated cardiovascular outcomes within rigorously followed trial populations, whereas Yun et al. [8] contribute nationwide real-world data with substantial statistical power and informative dose-response analyses. Second, this review places particular emphasis on temporal relationships and recurrence patterns, which are often underappreciated but highly relevant to causal interpretation. Third, by maintaining a specific focus on cardiovascular mortality and related cardiovascular outcomes, the review addresses a clinically important endpoint that has direct implications for risk assessment and management in diabetes care.

Limitations

Several limitations should be acknowledged when interpreting the findings of this review. A substantial proportion of the included evidence is derived from post hoc analyses of randomized trials, which, although methodologically robust in outcome ascertainment and follow-up, remain observational with respect to hypoglycemia as the exposure of interest. Residual confounding, therefore, remains a concern across all study designs, since hypoglycemia is more likely to occur in patients with advanced disease, insulin use, renal impairment, heart failure, or other features that independently increase cardiovascular risk. In addition, the potential for reverse causation cannot be excluded, whereby underlying clinical instability may predispose to both hypoglycemia and adverse cardiovascular outcomes. There was also variability across studies in the definitions of hypoglycemia, particularly for non-severe events, as well as heterogeneity in patient populations, baseline cardiovascular risk, and treatment context. Finally, although several plausible biological pathways support the observed associations, direct mechanistic confirmation in clinical settings remains limited, which constrains stronger inferences regarding causality.

Future Directions

Future research should move beyond secondary and post hoc analyses and prioritize prospective studies in which hypoglycemia is explicitly treated as the primary exposure of interest rather than a treatment-related byproduct. Such studies would benefit from more precise characterization of event timing, severity, recurrence, and clinical context [18]. The integration of continuous glucose monitoring data is likely to be especially valuable, as it would permit more accurate quantification of hypoglycemic burden, nocturnal episodes, and glycemic variability, all of which may influence cardiovascular risk but are incompletely captured by conventional reporting methods. Further work is also needed to clarify threshold effects, including whether risk is driven predominantly by severity, frequency, or a combination of both, and to identify particularly vulnerable phenotypes such as patients with heart failure, chronic kidney disease, autonomic neuropathy, or established atherosclerotic cardiovascular disease [20]. Importantly, future studies should aim to distinguish more clearly between hypoglycemia as a causal trigger and as a prognostic marker through robust adjustment strategies, advanced causal inference methods such as marginal structural models or mediation analyses, and, where feasible, interventional designs. A more refined understanding of these subgroups may help determine whether hypoglycemia functions primarily as a modifiable trigger, a prognostic marker, or both.

## Conclusions

The available evidence demonstrates a consistent association between hypoglycemic episodes, particularly severe events, and an increased risk of cardiovascular outcomes and mortality in individuals with diabetes. This relationship is supported across randomized trial-derived analyses and large observational cohorts, although variability in effect size and context underscores the complexity of interpretation. Current data suggest that hypoglycemia may act both as a short-term physiological stressor capable of precipitating adverse events and as a marker of underlying vulnerability in patients with advanced disease and higher baseline risk. While the included studies did not directly evaluate individualized glycemic target interventions, these findings support the clinical rationale for a more individualized and balanced therapeutic approach aimed at minimizing hypoglycemia while maintaining appropriate metabolic control. Recognizing hypoglycemia as a clinically meaningful signal rather than an isolated adverse event may enhance risk stratification and inform safer, more patient-centered diabetes management strategies.
